# Health-related quality of life in sarcoidosis patients and the effect of occupational exposures: a cross-sectional study

**DOI:** 10.1186/s12890-025-03552-w

**Published:** 2025-02-17

**Authors:** Denis Vinnikov, Leonid Strizhakov, Tatsiana Rybina, Sergey Babanov

**Affiliations:** 1 Al-Farabi Kazakh National University, 71 al-Farabi Avenue, Almaty, 050040 Kazakhstan; 2 Peoples’ Friendship University of Russia (RUDN University), Moscow, Russian Federation; 3 The Federal State Budgetary Scientific Institution “Izmerov Research Institute of Occupational Health”, Moscow, Russian Federation; 4 Tareev Clinic of Internal Diseases, Sechenov First Moscow State Medical University, Moscow, Russian Federation; 5 Department of Internal Diseases, Lomonosov Moscow State University, Moscow, Russian Federation; 6 National Centre of Occupational Safety and Health, Minsk, Belarus; 7 “MedEvery” Scientific and Practical Centre, Minsk, Belarus; 8 Department of Occupational Diseases and Clinical Pharmacology, Samara State Medical University, Samara, Russian Federation

**Keywords:** Occupational, Risk factors, Epidemiological, Questionnaire, Exposure

## Abstract

**Background:**

Health-related quality of life (HRQL) in patients with sarcoidosis has been related to treatment, symptoms, organ involvement and disease severity, but little is known about its association with occupation. The aim was to quantify HRQL in occupationally exposed sarcoidosis patients compared to their nonexposed counterparts.

**Methods:**

A total of 221 sarcoidosis patients (median age 49 years, interquartile range (IQR) 37–60 years) with a histologically confirmed diagnosis were recruited from university hospitals and outpatient centers in Belarus, Kazakhstan, and the Russian Federation. General (with SF-8) and specific (with K-BILD) HRQL were compared between patients who were ever exposed to 24 occupational factors and nonexposed patients in adjusted multivariable models.

**Results:**

Work in the office and office equipment (beta − 3.60 (95% confidence interval (CI) -6.91;-0.29)) was significantly associated with a poorer SF-8 physical component score (PCS) independent of sex, whereas exposure to irritant gases was strongly associated with a worse mental component score (MCS), adjusted for sex and smoking beta − 7.11 (95% CI -12.83;-1.39). Irritant gas (beta − 17.2 (95% CI -29.3;-5.1)) and work in the office (beta − 7.9 (95% CI -14.7;-1.0)) were associated with worse K-BILD total scores, while only the latter was associated with breathlessness and activities (BA) scores. Exposure to flour, irritant gas and office work were associated with the psychological (P) domain. Exposure to flour, irritant gas and work in the office could predict chest symptom (CS) scores.

**Conclusions:**

In patients with sarcoidosis and occupational exposure, patients may exhibit worse HRQL, but further research is needed to ascertain the interplay of individual and occupational factors.

**Supplementary Information:**

The online version contains supplementary material available at 10.1186/s12890-025-03552-w.

## Background

Sarcoidosis is believed to be a rare chronic disease with multiorgan involvement, but pulmonary sarcoidosis remains the most prevalent clinical manifestation [1]. Various treatment options exist, but patients in many studies have reported that in addition to symptoms and functional status, improvements in quality of life, including health-related quality of life (HRQL), may be the leading treatment goal for patients [2, 3]. Evidence-based options to improve QL are still lacking. HRQL is related to all aspects of sarcoidosis treatment and prognosis, including the side effects of medications that patients have to take for years, overall treatment efficacy and the presence of polypragmasia [4, 5]. Therefore, HRQL should be assessed at all stages of treatment and rehabilitation, and treatments should ideally improve HRQL, but the impact of treatment interventions on HRQL has yet to be ascertained [3, 6].

Furthermore, HRQL in patients with sarcoidosis may be related not only to treatment and clinical outcomes but also to risk factors and disease etiology. At present, sarcoidosis remains an idiopathic immunoreactive disease, but the contribution of selected environmental and occupational factors has long been under study. Exposure to vapors, gases, dust, and fumes in the workplace is often considered a risk factor or even an etiology of chronic respiratory diseases, including sarcoidosis. In the latter, occupational exposures were identified or suspected in 0–54% of cases, with a weighted meta-proportion of 30% (95% CI, 17–45%) [7]. The data from published studies were inconsistent and sometimes even differed in terms of the most known risk factors for sarcoidosis, such as firefighting, military service, working as a healthcare professional, and occupational exposure to metal and silica [8–11]. Since sarcoidosis affects people in the middle of their lives and is associated with diminished work ability [12], which is directly related to depression and fatigue, the most frequently reported symptoms of sarcoidosis, temporary or permanent disability and even job loss are directly related to the disease.

In addition, very little is known about whether patients with sarcoidosis exposed to suspected occupational risk factors have worse HRQL than their nonexposed counterparts. Because HRQL is considered a complex understating of personal well-being with regard to medical conditions, the occupational nature of exposure may also affect HRQL, but this hypothesis has not attracted close attention in the published literature. HRQL is usually assessed with patient-reported outcome measures, either general or specific for the disease [3, 6], which have identified an array of associations in sarcoidosis, including treatment interventions, income, rehabilitation, disease severity, clinical symptoms, and organ involvement. However, almost nothing is known whether HRQL is associated with occupation. We therefore planned this study to quantify HRQL, both generally and specifically, in occupationally exposed sarcoidosis patients and compare it with that of patients of likely unknown origin.

## Methods

### Study design

This study was approved by the Committee on Bioethics of the National Centre of Occupational Safety and Health (Belarus), al-Farabi Kazakh National University (Kazakhstan) and Sechenov First Moscow State Medical University (Russian Federation), and all participants provided informed consent to participate. We consecutively included patients with pulmonary sarcoidosis (identified as D86 using the ICD10), which we identified from hospital records. Diagnoses should have been verified at prior hospitalization at some time in the past, and a typical clinical presentation with histological confirmation was required for inclusion. All patients were 18 years and older. Patients were invited to participate during their hospitalization and from the outpatient centers from a larger case-control study [13], and patient recruitment lasted from November 2018 to May 2023. More information on patients’ profile can be found elsewhere [13]. All enrolled patients exhibited pulmonary sarcoidosis, but some also had extrapulmonary manifestations (22% patients also had skin, cardiac or neurological involvement alone or in combination). At the time of inclusion, 60% of patients did not receive treatment and were in remission, whereas 18% received corticosteroids and the remaining 22% were on methotrexate. Clinical trial number: not applicable.

### Questionnaire

All patients were offered a self-administered questionnaire in Russian following informed written consent. We first included questions on baseline demographic information, including birthdate, sex, permanent residence, total years of work, highest education level attained and detailed smoking status (never, ex-, current daily, current occasional conventional cigarette smokers); date of first diagnosis and exercise frequency (at least 3 times a week) were also queried. Because the aim of this presentation was to ascertain HRQL differences in those ever exposed in the workplace to the exposures of interest, we offered a detailed occupational exposure history questionnaire [14]. This tool was chosen because it allowed not only verification of ever-exposure but also identification of years of exposure and average duration of work week in hours. This tool was previously used in studies with idiopathic pulmonary fibrosis patients and specifically stressed exposures known to have an association with this wide group of fibrotic diseases. In particular, questions on ever exposure were listed for 24 selected occupational exposures, also detailing the period of exposure and the duration of the week in hours. The last question asked participants to specify a major lifetime occupation. An additional file shows this in more detail [see Additional file 1].

The HRQL part of the questionnaire included an 8-item short version of the SF tool, the SF-8, which offered one question for each of eight HRQL domains, including physical functioning, social functioning, role physical, role emotional, mental health, vitality, bodily pain and general health. The scores were recalculated as advised by the manual [15], that assigned a numerical score to each option in the answer. These eight domains were then grouped into two major components, the physical component score (PCS) and mental component score (MCS), of the general HRQL, which we analyzed in this study. The questionnaire is known for its excellent consistency and validity as an alternative to the initial wider 36-item tool and is extensively used in population-based studies [16–19].

The specific HRQL for patients with sarcoidosis in the present study was assessed with the K-BILD tool, which consists of 15 questions, eventually resulting in the total score (TS), breathlessness and activities (BA) score (4 questions out of 15), psychological (P) score (7 questions out of 15) and chest symptoms (CS) score (3 questions out of 15). Overall, a lower score was indicative of worse HRQL. This questionnaire has shown excellent internal consistency in previous studies [20].

### Statistical analysis

We first screened the data for normality using the Shapiro‒Wilk test, with most continuous variables showing a nonnormal distribution. Therefore, nonparametric tests were employed for the analysis. We present binary variables as frequencies or percentages, while continuous variables are summarized as medians with corresponding interquartile ranges (IQRs). The Mann‒Whitney U test was used for univariate comparisons of continuous variables, such as comparing men to women or exposed to nonexposed groups.

The primary outcomes of interest in this study were the SF-8 PCS and MCS scores and the K-BILD TS, BA, P and CS scores, which are expressed as continuous values. In addition to the studied exposures, we also tested the distribution of smoking, classified either as never- or ex- or current daily cigarette smoking; highest education; marital status; and overall work duration or years in service overall and stratified by sex. We first tested the association of sex, ever-smoking and each occupational exposure with each general and specific HRQL score, including PCS, MCS, K-BILD TS, BA, TS and CS using Mann-Whitney U-test. Given that there were 23 exposures tested (one exposure was excluded due to no exposed subjects), False Discovery Rate was considered and, therefore, we recalculated all crude p-values to adjusted values using the Benjamini-Hochberg procedure. Following univariate comparisons for each occupational exposure from the questionnaire, we identified those with significant associations and then included them in multivariable linear regression models adjusted for age, smoking status or sex, depending on the specific model, as indicated. All covariates were tested for collinearity with the variance inflation factor. In such models, all occupational exposures were treated as binary variables, as were smoking status and sex. The only continuous variable in our models was age. Furthermore, the reported beta coefficients with their 95% confidence intervals (CIs) are adjusted for all other included confounders. All the statistical tests were completed in NCSS 2024 (Utah, USA), and the p values of the tests were specified accordingly. In all analyses, p values less than 0.05 were considered significant.

## Results

The median age of our sample of sarcoidosis patients was 49 years, and men were significantly younger. Occupational exposure in the lifetime lasting for more than one year was reported by 59% of participants. There were only 12% daily smokers, most of whom were males (Table 1). Similarly, the majority of females were never smokers. We found that almost half of the sample had a college degree, which makes this sample representative of the general population in the three included countries. Furthermore, 77% were married at the time of inclusion. Overall, we found that the MCS score was greater than the PCS score in the entire sample, and women demonstrated worse general HRQL in both domains of this general instrument. Consistent with the general HRQL pattern, the K-BILD score, which is indicative of specific HRQL, was also significantly lower in women in all domains except the CS (Table 1).

**Table 1:** Baseline demographic portrait of the sample, the fraction of exposed patients and their HRQL scores

	Overall	Males	Females	*P*-value
N, %	221 (100)	112 (51)	109 (49)	-
Age, years	49 (37;60)	39 (33;53)	57 (48;64)	< 0.001
Education				
Secondary school	2 (1)	1 (1)	1 (1)	0.97
High school	33 (15)	16 (14)	17 (16)	
College	82 (37)	40 (36)	42 (39)	
University	99 (45)	52 (46)	47 (42)	
PhD degree	5 (2)	3 (3)	2 (2)	
Marital status				
Single	31 (14)	21 (19)	10 (9)	0.06
Married	170 (77)	84 (75)	86 (79)	
Divorced	20 (9)	7 (6)	13 (12)	
Cigarette smoking				
Never	124 (57)	32 (29)	92 (86)	< 0.001
Former	66 (31)	53 (49)	13 (12)	
Daily	26 (12)	24 (22)	2 (2)	
Any occupational exposure	130 (59)	71 (63)	59 (54)	0.16
SF-8 PCS	55.2 (48.7;64.7)	59.1 (50.2;64.7)	53.1 (48.0;61.3)	< 0.01
SF-8 MCS	59.1 (50.0;64.8)	60.8 (52.8;66.7)	56.0 (48.1;62.5)	< 0.01
K-BILD total score	85 (65;97)	92 (67;99)	79 (62;94)	< 0.01
K-BILD BA	23 (14;28)	24 (17;28)	18.5 (13;27)	< 0.01
K-BILD P	40 (31;43)	42 (31;44)	38 (30;42)	< 0.01
K-BILD CS	18 (14;21)	19 (15;21)	17.5 (13;21)	0.06

Note: medians of continuous variables are presented along with the interquartile range in brackets; PCS– physical component score; MCS– mental component score; BA– breathlessness and activities; P– psychological; CS– chest symptoms; p-values between males and females either from the χ2 test (for binary variables) or from the Mann‒Whitney U test (for continuous variables)

Overall, 59% (*N* = 130) were classified as ever-exposed. When exposed patients were compared with their non-exposed counterparts, neither sex composition, nor age, highest education attained or marital status differed between these two groups in the univariate comparisons (data not shown). However, daily smoking (16% vs. 5%) and former smoking (36% vs. 23%) were more prevalent in the exposed group, and there were more never-smokers in the non-exposed sarcoidosis patients’ group (71% vs. 48%), *p* < 0.001 from a 2*3 χ2-test. Exposed patients did not differ from the non-exposed group with regard to treatment (22% on steroids and 23% on methotrexate, and 16% and 20%, accordingly) and extrapulmonary involvement (20% and 23%).

The internal consistency of the eight SF-8 domains was high, and the Cronbach’s α was 0.90. When we first compared the SF-8 physical and mental component scores between those with any exposure and those with no exposure, we found no differences. However, in the univariate comparisons of 23 included occupational exposures, only employment in the office with printing equipment was associated with HRQL physical component (Table 2). In addition to this specific occupational exposure, physical component score was also associated with sex, but not with smoking cigarettes. When the mental component was considered, we found a somewhat different pattern in which a lower HRQL score was revealed in subjects exposed to irritant gases. as with physical component, males had higher MCS scores. Finally, ever smoking was associated with a worse mental HRQL but not with a worse physical component score. Moreover, we failed to identify any associations between any of the two component scores studied and age, years in service, marital status, highest attained education or alcohol consumption.

**Table 2:** SF-8 PCS and MCS scores in the univariate comparisons for 24 studied exposures (one exposure is not included due to no exposed subjects)

Factors	PCS			MCS		
	yes	no	*p*	yes	no	*p*
Occupational						
Sandblasting, grinding, milling or metal processing	56.5	55.2	0.61	58.1	59.1	0.85
Metal or steel production	63.5	54.8	0.53	52.6	59.1	0.74
Welding	59.1	54.7	0.65	58.8	59.1	0.97
Stone dust	54.4	55.1	0.98	56.6	59.1	0.69
Carbon or graphite powder	48.6	55.2	0.62	59.1	59.1	0.99
Glass wool	61.6	54.7	0.43	61.8	59.0	0.86
Ceramics	53.0	55.0	0.46	61.6	59.0	0.92
Asbestos or asbestos-containing products	58.3	55.0	0.65	62.2	59.0	0.92
Poultry	59.7	55.0	0.67	51.2	59.1	0.75
Hay or straw	55.0	55.0	0.86	58.9	59.1	0.91
Grain or flour	49.1	55.3	0.40	54.8	59.1	0.92
Timber or wood shavings	60.0	54.8	0.46	57.5	59.1	0.84
Paper production	53.4	55.1	0.84	51.3	59.1	0.58
Natural or artificial fibers	58.6	55.2	0.84	61.3	59.0	0.94
Ionizing radiation	56.9	55.2	0.96	54.5	59.1	0.89
Solvents	55.4	55.2	0.69	61.2	59.0	0.98
Open fire	64.7	55.0	0.54	62.2	59.0	0.97
Car or engine exhausts	54.9	55.2	0.94	57.0	59.1	0.87
Irritating gas	48.6	55.8	0.43	48.1	59.4	0.18
Lubricating oils	58.6	55.2	0.83	58.7	59.1	0.90
Ultrafast glue (cyanoacrylate)	58.5	55.0	0.63	62.3	59.0	0.88
Secondhand smoke in the workplace	52.6	55.6	0.42	57.5	59.8	0.84
Office copying and printing equipment	52.5	57.1	0.12	55.3	57.0	0.79
Male sex	59.1	53.1	< 0.01	60.9	56.0	< 0.01
Ever-smoking	56.0	54.3	0.11	57.7	60.3	0.02

Note: PCS– physical component score; MCS– mental component score. The median scores for the PCS and MCS are given. P-values were computed using Mann‒Whitney U test and then adjusted using Benjamini-Hochberg procedure for multiple comparisons

Despite no significant associations of occupational exposures with a physical component, we still tested work in the office adjusted for sex, because this exposure had the least unadjusted p-value (0.005), with this HRQL component. It was significantly associated with poorer physical component independent of sex. The beta coefficient for this exposure was − 3.60 (95% CI -6.91; -0.29) and − 3.30 (95% CI -5.85; -0.76) for females. When the MCS was adjusted for sex and ever-smoking, exposure to irritant gases was consistently and strongly associated with a worse mental component, beta − 7.11 (95% CI -12.83;-1.39).

The internal consistency of the K-BILD three domains was high, with a Cronbach’s α of 0.90. The K-BILD total score demonstrated some association with greater exposure in the univariate comparisons. Total score was associated with work with irritant gas and in the office, and some borderline association was found for work with grain or flour (Table 3). Breathlessness and activities domain did not exhibit any strong associations, whereas psychological domain scores were associated with irritant gases and in the office, and some borderline association was identified for work with grain or flour. A worse chest symptom score was associated with an occupational history of office work. In addition, the total score exhibited a significant association with sex (median in females 79 vs. 92 in males), smoking status (median 92 vs. 79 in never-smokers) and age (beta coefficient − 0.34 (95% CI -0.14; 95% CI -0.55)). Similar associations persisted for breathlessness and psychological scores. chest symptoms domain was associated with age (beta − 0.06 (95% CI -0.1; -0.01)).

**Table 3:** K-BILD total score along with three domains in the univariate comparisons for the 23 studied exposures (one exposure is not included due to no exposed subjects)

Occupational factors	Total			BA			*P*			CS		
	yes	no	*p*	yes	no	*p*	yes	no	*p*	yes	no	*p*
Sandblasting, grinding, milling or metal processing	86.5	85	0.97	24	22	0.69	38.5	40	0.81	20	18	0.99
Metal or steel production	92	83	0.67	27	22	0.53	41	40	0.81	19	18	0.99
Welding	90	83	0.99	24.5	22	0.72	41	40	0.98	18.5	18	0.94
Stone dust	86.5	85	0.98	26.5	22	0.83	41	40	0.81	19.5	18	0.99
Carbon or graphite powder	59	85	0.85	13	23	0.62	27	40	0.72	13	18	0.99
Glass wool	87	85	0.95	25	23	0.80	39.5	40	0.78	18	18	0.93
Ceramics	94	83	0.87	27	22.5	0.64	41	40	0.86	18	18	0.99
Asbestos or asbestos-containing products	81	85	0.98	25.5	23	0.71	33	40	0.81	18.5	18	0.99
Poultry	83	85	0.99	24	23	0.81	39	40	0.79	17	18	0.96
Hay or straw	67	86	0.73	19	23	0.61	35	40	0.38	16	18	0.99
Grain or flour	61	87	0.13	14.5	23	0.41	26.5	40	0.07	12.5	18	0.18
Timber or wood shavings	95	83	0.99	27.5	22	0.46	42.5	40	0.81	18.5	18	0.99
Paper production	68.5	85	0.81	17	23	0.75	35	40	0.56	15.5	18	0.99
Natural or artificial fibers	90	84	0.99	24	22.5	0.79	37	40	0.87	19	18	0.97
Ionizing radiation	60	85	0.72	15	23	0.63	28	40	0.74	14	18	0.99
Solvents	83	86	0.92	24	22.5	0.99	38	40	0.75	18	17	0.96
Open fire	95	83	0.22	27	22	0.46	43	40	0.15	19	18	0.99
Car or engine exhausts	88	84	0.97	26	22	0.69	38	40	0.85	17	18	0.99
Irritating gas	62	87.5	0.04	14	23	0.32	28	40	0.04	12	18	0.15
Lubricating oils	89	84	0.94	27	22	0.52	41	40	0.87	20	18	0.99
Ultrafast glue (cyanoacrylate)	83	85	0.99	28	22.5	0.54	36	40	0.78	21	18	0.99
Secondhand smoke in the workplace	79	87.5	0.20	19	24	0.46	39	40	0.32	17	18	0.99
Office copying and printing equipment	67	90	0.02	17	24	0.14	35	41	0.04	15	11	0.02

Note: BA– breathlessness and activities; P– psychological; CS– chest symptoms. P-values were computed using Mann‒Whitney U test and then adjusted using Benjamini-Hochberg procedure for multiple comparisons

Multivariable adjusted modeling of each domain yielded fewer significant lifetime exposures as predictors of worse specific HRQL. Thus, irritant gas (beta − 17.2 (95% CI -29.3;-5.1)) and work in the office (beta − 7.9 (95% CI -14.7;-1.0)) were associated with worse total scores (R^2^ for the model 0.18). When breathlessness score was considered an outcome in the adjusted model, only work in the office remained significant (beta − 3.0 (95% CI -5.4;-0.7)) (R^2^ for the model 0.16). Exposure to flour (beta − 7.0 (95% CI -13.9;-0.1)), irritant gas (beta − 9.4 (95% CI -14.6;-4.2)) and employment in the office (beta − 2.9 (95% CI -5.8;-0.1)) were negatively and significantly associated with the psychological domain (R^2^ for the model 0.17). Finally, we confirmed the effect of only three exposures for symptoms, both putting patients at risk of worse HRQL: exposure to flour (beta − 4.4 (95% CI -7.8;-0.9)), irritant gas (beta − 3.4 (95% CI -6.0;-0.8)) and work in the office (beta − 1.8 (95% CI -3.2;-0.3)), whereas these variables together with age explained 14% of the entire CS variability.

## Discussion

This study was planned to test the hypothesis that sarcoidosis patients with occupational exposure in their lifetime may have somewhat different HRQL than those with no known occupational history. We previously published no such studies, although HRQL in patients with sarcoidosis with regard to other predictors has been extensively examined elsewhere [3]. In our sample of 221 patients with histologically confirmed diagnoses, we elucidated some significant associations with occupational history. Work in the office with equipment has been consistently associated with poorer PCS of general HRQL and with all four domains of specific HRQL via K-BILD, whereas exposure to irritant gases in the past showed a negative correlation with SF-8 MCS and K-BILD TS, P and CS. In addition, exposure to flour also had some negative associations with the selected domains, and all these associations existed independent of smoking, age and sex. Taken together, these findings indicate that occupational history in patients with sarcoidosis has been overlooked and poorly understood in terms of its impact on HRQL, and the associations we have identified can help shape more targeted rehabilitation programs for these patients.

The landscape of earlier publications on environmental and occupational risk factors for sarcoidosis was wide enough and included an array of suspected risk factors from the workplace, including crystalline silica, work in agriculture and farming, exposure to birds, metals, firefighting and military service. Occupational exposures in sarcoidosis patients have gained the attention of researchers mostly in Western countries, but very little is known about the prevalence of exposure and the risk in countries of the former Soviet Union, where research on sarcoidosis has focused on treatment and prevention rather than risk factors. Our systematic review of Russian literature from former Soviet countries highlighted this gap and demonstrated that very few studies have been published and that many had a high potential for misclassification [8]. In the current study, we attempt to fill this gap and add more evidence on the association of suspected occupational exposures with the disease and the associated HRQL.

Most associations with occupational exposures before were inconsistent across published studies, but none of these studies addressed HRQL in patients with suspected occupational origin compared to those who had never been in contact with the agents or exposures of interest. However, HRQL in patients with sarcoidosis has been studied with regard to various treatments, such as corticosteroids [21, 22], methotrexate [23], infliximab and its newer substitutes [24, 25]; impact on daily life [2]; symptom severity [26]; and specific patient characteristics. Because none of the preceding studies attempted to quantify HRQL in occupationally exposed sarcoidosis patients compared with those with known no exposure, our study is indeed somewhat novel. We conclude that occupational exposure can worsen selected domains of HRQL, particularly in those with a past history of irritant gas exposure, and must be considered when patient-reported outcome measures are evaluated in the clinical process as factors explaining poor reported scores.

As a recent review revealed [3], a wide range of tools are available for HRQL estimation in sarcoidosis patients, including the Sarcoidosis Health Questionnaire [27], the SAT fatigue module [28] and King’s Sarcoidosis Questionnaire [29]. Nevertheless, we elected K-BILD as a tool in our study because it could still be used in a broader group of patients with any interstitial lung disease, including sarcoidosis, and because it has been successfully used in our clinics since its introduction for patient management. This study showed the excellent consistency of this tool with the general SF-8 in its capacity to identify poorer outcomes. Both general and specific tools t were consistent in reporting poorer HRQL in patients exposed to irritant gases and working in the office with printing equipment.

The use of the general HRQL tool, the SF-8, in this study allows us to compare our HRQL scores with those of the general population in Kazakhstan, whereas population-based studies reporting scores in the general population in the Russian Federation and Belarus in recent years are lacking. We previously reported median PCS and MCS scores for Almaty of 57.7 and 59.5, respectively [16], and the adjusted beta coefficients for women were − 2.19 and − 3.38 for the PCS and MCS, respectively. According to the current presentation of sarcoidosis patients, the median scores were 55.2 and 59.9, indicating somewhat worse HRQL in these patients than in the general population sample and the difference was even greater when compared to selected working population in Almaty [30]. This is consistent with other studies in sarcoidosis patients elsewhere, but the difference with the general population in our case was not that pronounced. We believe that specific tools such as the K-BILD could be more sensitive for identifying specific health problems in this group that are directly related to dyspnea, fatigue and general health status.

Associations identified in our presentation need deeper insight into their possible pathophysiology. We found no direct assessments of the association of occupational exposure to irritant gases in the literature with HRQL, even in healthy working populations, which could explain the association we identified. Nevertheless, some inferences can be drawn from environmental and occupational air pollution with selected gases, such as sulfur dioxide. This pollutant was found to reduce HRQL in a population-based study in Korea [31], but in other reports, the association was not significant [32]. More robust evidence has been obtained for sulfur-containing chemical weapons, such as sulfur mustard [33–35]. Exposure to this warfare was found to dramatically reduce all domains of HRQL, indicating the strong association of sulfur with HRQL. The association we found was strong enough to be retained in the multivariable analysis and adjusted for all other exposures. However, further in-depth insight into the toxicological mechanisms underlying this effect is needed. The implications for public health, however, are quite clear and include expected poorer outcomes from treatment and rehabilitation in such patients with specific occupational exposure to irritant gases.

The major limitation of our study was the potential misclassification of exposure resulting from the use of a subjective self-report questionnaire, and the data to prove or support reported exposures from employers, central biobanks or other databases were not available. We also understand that some differential misclassification of exposure cannot be completely ruled out [36]. The limited range of environmental exposures and other confounders in our questionnaire, which were prompted by previous research and the specific questionnaire that we had chosen, could cause us to miss other underrepresented exposures, which also represents a limitation. We also recognize that some unmeasured confounding factors were present. In addition, because this study was completed on sarcoidosis patients with no healthy controls, the associations we observed for the occupational exposures may affect everyone and not sarcoidosis patients only. Finally, the cross-sectional design employed in this study can reveal only this association, but causality should be further verified in cohort observations.

In addition to the limitations mentioned above, a healthy worker selection bias may also be present in the sample of sarcoidosis patients and thus bias the effect. Given that patients with a respiratory disease may not feel fit enough for employment in workplaces with chemical exposures, such as with irritant gases, but may be still be fit to work in the office, we then see a reduced score for HRQL in those exposed to officer work. This healthy worker pre-employment effect is known to exhibit better health attributes in subjects working with hazards, as we also showed before in the mining industry [37, 38]. Healthy worker effect may affect subjects in a discrepant fashion with regard to race, type of industry, sex, smoking status, etc., and thus uncertainty still remains how known effect will influence the association [39]. Because the proposed approach to combat the bias is the inclusion of the same population at the same time point without the exposure of interest [40], we expect the least bias in our sample, but predicting the direction and magnitude of this effect is still challenging.

## Conclusions

In conclusion, our study is the first to shed some light on the association of HRQL with likely occupational factors and to compare it with that of patients who have never been exposed. We have now demonstrated that HRQL in sarcoidosis patients with specific occupational exposures in the past, including irritant gases and office work with printing equipment and even exposure to flour, could demonstrate worse scores. While our findings provide valuable insights into potential risk factors for worse HRQL in this specific population, we call for further research to better understand the complex interplay of occupational exposures and other variables in the way patients report their outcomes, which they consider important.

## Electronic supplementary material

Below is the link to the electronic supplementary material.

Supplementary Material 1

## Data Availability

All data generated or analysed during this study are included in this published article.

## Abbreviations

BA: Breathlessness and activities
CI: Confidence interval
CS: Chest symptom
HRQL: Health-related quality of life
IQR: Interquartile range
MCS: Mental component score
P: Psychological
PCS: Physical component score
TS: Total score
