# Supplementary material for: Health-related quality of life in sarcoidosis patients and the effect of occupational exposures: a cross-sectional study
Source: BMC Pulm Med. 2025 Feb 17;25:83. doi: 10.1186/s12890-025-03552-w (PMC11834176; doi:10.1186/s12890-025-03552-w)
Supplement: Supplementary file 1 — Supplementary Material 1 [file 12890_2025_3552_MOESM1_ESM.pdf]

Dear participant of the study on the occupational risk factors for sarcoidosis. We kindly ask you to think over each question carefully and respond to each one in a candid manner.

1. Surname, name____
2. Year of birth ____ 3. Sex _____ 4. Total years in service at a time of the survey ______________

5. Highest education:
 □ Secondary school
 □ High school
 □ College
 □ University degree
 □ Academic degree (PhD, etc.)

6. Marital status:
 □ Single
 □ Married/partnered
 □ Divorced

7. Have you ever smoked cigarettes?
 □ No (tick and skip to question #12)
 □ Yes
8. Do you continue smoking at present?
 □ No (tick and skip to question #12)
 □ Yes
9. Do you smoke cigarette daily?
 □ No (tick and skip to question #12)
 □ Yes
10. How many cigarettes a day do you smoke? _______
11. For how many years have you been smoking up to now? ________
12. Are you engaged in any sport or leisure physical activity off work for at least 3 times a week?
 □ No
 □ Yes
13.How many times a week are you engaged in any sport or leisure physical activity off work _______________
14.Please chose the option which best corresponds to your alcohol consumption level:
 □ Never
 □ Sometimes, small amount (less than once a week, no more than one glass of wine)
 □ Sometimes, moderate amount (less than once a week)
 □ At least once a week
15. Have you ever been working in sandblasting, grinding, milling or metal processing?
 □ No If yes, when did you start and leave? From ______ year till ______ year
 □ Yes How many hours a week on average? __________________
16. Have you ever been working in metal or steel production?
 □ No If yes, when did you start and leave? From ______ year till ______ year
 □ Yes How many hours a week on average? __________________
17. Have you ever been working with welding?
 □ No If yes, when did you start and leave? From ______ year till ______ year
 □ Yes How many hours a week on average? __________________
18. If you answered “Yes” to any of the three questions above, please provide more information on that exposure:

	Yes	No	Do not know
18.1 Iron/steel			
18.2 Stainless steel			
18.3 Copper			
18.4 Aluminum			
18.5 Brass			
18.6 Bronze			
18.7 Hard metal			
18.8 Tungsten, cobalt			
18.9 Galvanized sheet			
18.10 Dyed metal			
18.11 Silver/gold			

18.12 Tin			
18.13 Lead			
18.14 Cadmium			

19. Have you ever been working in nickel or chrome plating?
□ No If yes, when did you start and leave? From ______ year till ______ year
□ Yes How many hours a week on average? ___________________

20. Have you ever been working with stone dust (In a quarry, mine, with building demolition, in the foundry, with a furnace)?
□ No If yes, when did you start and leave? From ______ year till ______ year
□ Yes How many hours a week on average? ___________________

21. Have you ever been working with carbon or graphite powder?
□ No If yes, when did you start and leave? From ______ year till ______ year
□ Yes How many hours a week on average? ___________________

22. Have you ever been working with glass wool?
□ No If yes, when did you start and leave? From ______ year till ______ year
□ Yes How many hours a week on average? ___________________

23. Have you ever been working with ceramics?
□ No If yes, when did you start and leave? From ______ year till ______ year
□ Yes How many hours a week on average? ___________________

24. Have you ever been working with asbestos or asbestos-containing products?
□ No If yes, when did you start and leave? From ______ year till ______ year
□ Yes How many hours a week on average? ___________________

25. Have you ever been working with poultry (parrots, pigeons)?
□ No If yes, when did you start and leave? From ______ year till ______ year
□ Yes How many hours a week on average? ___________________

26. Have you ever been working with hay or straw?
□ No If yes, when did you start and leave? From ______ year till ______ year
□ Yes How many hours a week on average? ___________________

27. Have you ever been working with grain or flour?
□ No If yes, when did you start and leave? From ______ year till ______ year
□ Yes How many hours a week on average? ___________________

28. Have you ever been working with timber or wood shavings?
□ No If yes, when did you start and leave? From ______ year till ______ year
□ Yes How many hours a week on average? ___________________

29. Have you ever been working in the paper production (with paper dust)?
□ No If yes, when did you start and leave? From ______ year till ______ year
□ Yes How many hours a week on average? ___________________

30. Have you ever been working with natural (wool, cotton, etc.) or artificial (nylon, viscose) fibers?
□ No If yes, when did you start and leave? From ______ year till ______ year
□ Yes How many hours a week on average? ___________________

31. Have you ever been working with ionizing radiation?
□ No If yes, when did you start and leave? From ______ year till ______ year
□ Yes How many hours a week on average? ___________________

32. Have you ever been working with solvents (acetone, White spirit, toluene, trichloroethylene)?
□ No If yes, when did you start and leave? From ______ year till ______ year
□ Yes How many hours a week on average? ___________________

33. Have you ever been working with open fire (as a firefighter)?
□ No If yes, when did you start and leave? From ______ year till ______ year
□ Yes How many hours a week on average? ___________________

34. Have you ever been working with car or engine exhausts (in a repair shop or garage)?
□ No If yes, when did you start and leave? From ______ year till ______ year
□ Yes How many hours a week on average? ___________________

35. Have you ever been working with an irritating gas (ammonia, chlorine oxides, sulfur oxides)?
□ No If yes, when did you start and leave? From ______ year till ______ year
□ Yes How many hours a week on average? ___________________

36. Have you ever been working with lubricating oils?
□ No If yes, when did you start and leave? From ______ year till ______ year
□ Yes How many hours a week on average? ___________________

37. Have you ever been working with ultrafast glue (cyanoacrylate)?
□ No If yes, when did you start and leave? From ______ year till ______ year

□ Yes How many hours a week on average? ___________________

38. Have you ever been exposed to secondhand smoke in the workplace?

□ No If yes, when did you start and leave? From ______ year till ______ year

□ Yes How many hours a week on average? ___________________

39. Have you ever been working with office copying and printing equipment?

□ No If yes, when did you start and leave? From ______ year till ______ year

□ Yes How many hours a week on average? ___________________

40. Please list your occupations in which you worked for at least 2 years:

____

41. Please choose one occupation from this list in which you worked for most in your lifetime:

47.1. Pilot
47.2. Pharmacist
47.3. Teacher/lecturer at school/college/university
47.4. Gym coach/instructor
47.5. Law enforcement officer
47.6. Medical
47.7. Waiter, barman
47.8. Printing house staff
47.9. Public transport driver
47.10. Cleaner
47.11. Cashier
47.12. Rescuer/firefighter
47.13. Catering personnel
47.14. Construction
47.15. Water supply/sewage
47.16. Librarian
47.17. Security officer
47.18. Bank personnel
47.19. Military
47.20. Lawyer
47.21 Engineer
47.22 Businessman/manager

SF-8:

1. Overall, how would you rate your health during the past one week?

□ Excellent
□ Very good
□ Good
□ Fair
□ Poor
□ Very poor

2. During the past one week, how much did physical health problems limit your usual physical activities (such as walking or climbing stairs)?

□ Not at all
□ Very little
□ Somewhat
□ Quite a lot
□ Could not do physical activities

3. During the past one week, how much difficulty did you have doing your daily work, both at home and away from home, because of your physical health?

□ None at all
□ A little bit
□ Some
□ Quite a lot
□ Could not do daily work

4. How much bodily pain have you had during the past one week?

□ None
□ Very mild
□ Mild
□ Severe
□ Very severe

5. During the past pone week, how much energy did you have?

□ Very much
□ Quite a lot
□ Some
□ A little
□ None

6. During the past one week, how much did your physical health or emotional problems limit your usual social activities with family of friends?

□ Not at all
□ Very little
□ Somewhat
□ Quite a lot
□ Could not do social activities

7. During the past one week, how much have you been bothered by emotional problems (such as feeling anxious, depressed or irritable)?

□ Not at all
□ Slightly
□ Moderately
□ Quite a lot
□ Extremely

8. During the past one week, how much did personal or emotional problems keep you from doing your usual work, school or other daily activities?

□ Not at all
□ Very little
□ Somewhat
□ Quite a lot
□ Could not do daily activities

K-BILD

1. In the last 2 weeks, I have been breathless climbing stairs or walking up an incline or hill.
1) Every time
2) Most times
3) Several times
4) Some times
5) Occasionally
6) Rarely
7) Never

2. In the last 2 weeks, because of my lung condition, my chest has felt tight.
1) All of the time
2) Most of the time
3) A good bit of the time
4) Some of the time
5) A little of the time
6) Hardly any of the time
7) None of the time

3. In the last 2 weeks have you worried about the seriousness of your lung complaint?
1) All of the time
2) Most of the time
3) A good bit of the time
4) Some of the time
5) A little of the time
6) Hardly any of the time
7) None of the time

4. In the last 2 weeks have you avoided doing things that make you breathless?
1) All of the time
2) Most of the time
3) A good bit of the time
4) Some of the time
5) A little of the time
6) Hardly any of the time
7) None of the time

5. In the last 2 weeks have you felt in control of your lung condition?
1) None of the time
2) Hardly any of the time
3) A little of the time
4) Some of the time
5) A good bit of the time
6) Most of the time
7) All of the time

6. In the last 2 weeks, has your lung complaint made you feel fed up or down in the dumps?
1) All of the time
2) Most of the time
3) A good bit of the time
4) Some of the time
5) A little of the time
6) Hardly any of the time
7) None of the time

7. In the last 2 weeks, I have felt the urge to breathe, also known as ‘air hunger’?
1) All of the time
2) Most of the time
3) A good bit of the time
4) Some of the time
5) A little of the time
6) Hardly any of the time
7) None of the time

8. In the last 2 weeks, my lung condition has made me feel anxious:
1) All of the time
2) Most of the time
3) A good bit of the time
4) Some of the time
5) A little of the time
6) Hardly any of the time
7) None of the time

9. In the last 2 weeks, how often have you experienced ‘wheeze’ or whistling sounds from your chest?
1) All of the time
2) Most of the time
3) A good bit of the time
4) Some of the time
5) A little of the time
6) Hardly any of the time
7) None of the time

10. In the last 2 weeks, how much of the time have you felt your lung disease is getting worse?
1) All of the time
2) Most of the time
3) A good bit of the time
4) Some of the time
5) A little of the time
6) Hardly any of the time
7) None of the time

11. In the last 2 weeks has your lung condition interfered with your job or other daily tasks?
1) All of the time
2) Most of the time
3) A good bit of the time
4) Some of the time
5) A little of the time
6) Hardly any of the time
7) None of the time

12. In the last 2 weeks have you expected your lung complaint to get worse?
1) All of the time
2) Most of the time
3) A good bit of the time
4) Some of the time
5) A little of the time
6) Hardly any of the time
7) None of the time

13. In the last 2 weeks, how much has your lung condition limited you carrying things, for example, groceries?
1) All of the time
2) Most of the time
3) A good bit of the time
4) Some of the time
5) A little of the time
6) Hardly any of the time
7) None of the time

14. In the last 2 weeks, has your lung condition made you think more about the end of your life?
1) All of the time
2) Most of the time
3) A good bit of the time
4) Some of the time
5) A little of the time
6) Hardly any of the time
7) None of the time

15. Are you financially worse off because of your lung condition?
1) A significant amount
2) A large amount
3) A considerable amount
4) A reasonable amount
5) A small amount
6) Hardly at all
7) Not at all
